# Intranasal Mometasone After External Dacryocystorhinostomy—Necessity or Convention? A Retrospective Two-Centre Cohort Study

**DOI:** 10.3390/jcm15218505

**Published:** 2026-11-02

**Authors:** Leticia Ortega-Evangelio, Gonzalo Roig-Ferreruela, Cristina Peris-Martínez

**Affiliations:** 1 Department of Ophthalmology, Fundación Oftalmológica Médica de la Comunidad Valenciana (FOM), 46015 Valencia, Spaincristina.p.peris@uv.es (C.P.-M.); 2 Department of Oculoplastics, Clínica Baviera, 46005 Valencia, Spain; 3 Surgery Department, Ophthalmology, School of Medicine, University of Valencia, Av. Blasco Ibáñez 15, 46010 Valencia, Spain; 4 Spanish Network of Inflammatory Diseases: REI-RICORS (RD24/0007/0004) of the Institute of Health Carlos III (ISCIII), Spanish Government, 28029 Madrid, Spain

**Keywords:** dacryocystorhinostomy, external dacryocystorhinostomy, nasolacrimal duct obstruction, intranasal corticosteroids, mometasone furoate, postoperative care, epiphora

## Abstract

**Background/Objectives:** Intranasal corticosteroids are frequently prescribed after dacryocystorhinostomy (DCR) to modulate mucosal healing and prevent ostium closure, but evidence supporting their use after external DCR (EXT-DCR) is scarce. This study compared early anatomical success after EXT-DCR under two centre-specific postoperative protocols that differed in the use of intranasal mometasone. **Methods:** Retrospective comparative cohort study of 243 consecutive EXT-DCR procedures (211 patients) for distal lacrimal obstruction performed by a single surgeon using the same technique at two centres between 2019 and 2025. Postoperative treatment was determined by each centre’s standing protocol: both included oral amoxicillin–clavulanic acid for one week, and one centre additionally prescribed intranasal mometasone furoate twice daily for two months (mometasone group, *n* = 64; control group, *n* = 179). The primary outcome was anatomical success, defined as lacrimal patency on irrigation at two months. Groups were compared with Fisher’s exact test and a multivariable logistic regression adjusted for age and sex. **Results:** Anatomical success was 93.8% (60/64) in the mometasone group and 89.9% (161/179) in the control group (absolute difference 3.8 percentage points; 95% CI −5.7 to 10.3; *p* = 0.45). After adjustment for age and sex, treatment group was not significantly associated with success (control vs. mometasone: OR 0.57; 95% CI 0.18–1.77; *p* = 0.33). No statistically significant associations were detected for age or sex. **Conclusions:** In this cohort, adding a two-month course of intranasal mometasone to standard postoperative care after EXT-DCR was not associated with a statistically significant improvement in early anatomical success. Given the limited statistical power and a confidence interval that does not exclude a clinically meaningful benefit, this absence of a significant difference does not establish therapeutic equivalence. Adequately powered prospective trials with longer follow-up are needed before routine intranasal corticosteroid use after EXT-DCR can be either recommended or abandoned.

## 1. Introduction

Dacryocystorhinostomy (DCR) is the standard surgical treatment for primary acquired nasolacrimal duct obstruction (PANDO). It creates a mucosa-lined anastomosis between the lacrimal sac and the nasal cavity through an osteotomy and can be performed through an external (EXT-DCR) or an endonasal endoscopic (END-DCR) approach [1]. Both approaches achieve high success rates. In a recent systematic review and meta-analysis, EXT-DCR showed a pooled success rate of 91.6%, significantly higher than that of endoscopic laser-assisted DCR (80.3%) [2], whereas non-laser mechanical END-DCR has achieved results comparable to those of EXT-DCR in several series [3,4]. The choice of technique therefore depends on surgeon expertise, lacrimal and nasal anatomy, and patient preference [5].

Postoperative management after DCR varies widely and commonly combines topical ocular antibiotics and corticosteroids, intranasal corticosteroids, intranasal antibiotics, nasal decongestants, and oral antibiotics [6]. The rationale for intranasal corticosteroids is biological: most failures result from fibrosis, cicatricial closure, or granulation tissue at the ostium during the first postoperative months [7,8], and corticosteroids suppress inflammation and fibroblast activity during this period. In a series of 100 anatomically failed procedures, cicatricial closure of the ostium was found in approximately half of failed EXT-DCRs, together with inadequate osteotomy and sac marsupialization [9], and patient-related factors such as postoperative soft-tissue infection, previous facial trauma, and acute dacryocystitis also influence the outcome of external lacrimal surgery [10]. Evidence supporting intranasal corticosteroids comes almost exclusively from END-DCR, where steroid-impregnated nasal packing has been associated with fewer ostium granulations and better functional outcomes [11,12,13] and topical intranasal steroids have been used to manage postoperative granulomas [14].

For EXT-DCR, the evidence is even more limited. In their systematic review and meta-analysis of post-surgical therapies, Vinciguerra et al. could not perform a statistical analysis of postoperative medical treatment after EXT-DCR because too few eligible studies were available [6]. As a result, intranasal corticosteroids continue to be prescribed after EXT-DCR largely by convention, despite their cost, the burden of prolonged multiple daily applications, and a possible effect on adherence to the remaining postoperative regimen.

The aim of this study was to compare early anatomical success after EXT-DCR under two centre-specific postoperative protocols that differed in the use of intranasal mometasone furoate, in a cohort operated on by a single surgeon using a standardized technique.

## 2. Materials and Methods

### 2.1. Study Design and Setting

This was a retrospective comparative cohort study of consecutive patients who underwent EXT-DCR between June 2019 and September 2025 at two centres in Valencia, Spain: FOM, a public centre (no intranasal corticosteroid; procedures performed between 2019 and 2025), and Clínica Baviera, a private centre (mometasone protocol; procedures performed between 2021 and 2025). All procedures were performed by the same surgeon (L.O.-E.) using the same technique. The study was reported following the Strengthening the Reporting of Observational Studies in Epidemiology (STROBE) guidelines [15].

The study adhered to the tenets of the Declaration of Helsinki and, given its retrospective design, received exemption approval from the institution’s legal and ethics committee. Written informed consent for surgery was obtained from all patients.

### 2.2. Participants

Medical records of all consecutive patients who underwent EXT-DCR during the study period were reviewed. Patients were included if they had a distal lacrimal drainage obstruction (nasolacrimal duct obstruction) confirmed by lacrimal irrigation. Patients with proximal obstruction (punctal, canalicular, or common canalicular) were excluded. The number of records screened and excluded for proximal obstruction was not recorded. A total of 243 procedures in 211 patients were included in the analysis. Thirty-two patients underwent staged bilateral EXT-DCR on different dates (26 in the control group and 6 in the mometasone group). Both procedures in each of these patients were performed at the same centre and therefore under the same postoperative protocol, and each procedure was analysed as a separate observation. Fifteen procedures were revision DCRs after a previous DCR on the same side (4 in the mometasone group and 11 in the control group). All patients attended the 2-month assessment, and no procedure was excluded because of missing outcome data.

### 2.3. Surgical Technique

All procedures were performed under locoregional anaesthetic block with intravenous sedation. A skin incision was made 10 mm from the medial canthal tendon with a cold scalpel. Blunt dissection was carried out in layers, retracting the orbicularis muscle, down to the superficial limb of the medial canthal tendon, which was then divided. After exposure of the lacrimal fossa, the osteotomy was created with a powered osteotome. The lacrimal sac was tented with a lacrimal probe, an anterior flap was fashioned from its medial wall, and the posterior portion was excised with Westcott scissors. The nasal mucosa was then opened and a bicanalicular silicone tube was placed. The anterior lacrimal sac flap was sutured to the anterior nasal mucosal flap, and the orbicularis muscle was closed. The skin was closed with tissue adhesive except when wound apposition was difficult [16]. Nasal packing was placed at the end of surgery and removed after 48 h. The silicone tube was removed two months after surgery.

### 2.4. Exposure: Postoperative Treatment

Postoperative treatment was not chosen by the surgeon for individual patients; it was dictated by the standing postoperative protocol of each centre. These protocols had been established by each institution before the study period and were applied to all patients operated on there; the surgeon followed the protocol of the centre in which each procedure took place and did not modify it according to patient characteristics. The centre in which each patient underwent surgery depended on the patient’s healthcare coverage (private or public) and referral pathway, not on the clinical features of the obstruction. In both centres, patients received oral amoxicillin–clavulanic acid 875/125 mg every 8 h for 7 days, and no topical ocular drops of any kind were prescribed. At Clínica Baviera, patients additionally received intranasal mometasone furoate nasal spray (50 µg per spray) one spray into the operated-side nostril twice daily (total daily dose, 100 µg), starting after removal of the nasal packing at 48 h, for two months (mometasone group); treatment was therefore discontinued at the 2-month visit, coinciding with silicone tube removal and outcome assessment. At FOM, no intranasal corticosteroid was prescribed (control group). No other postoperative medication, nasal irrigation, or endonasal debridement was routinely used in either centre. Adherence to intranasal mometasone was not assessed.

### 2.5. Outcomes and Variables

The primary outcome was anatomical success, defined as free passage of saline into the nose without reflux on irrigation through the inferior punctum at the 2-month visit, after removal of the silicone tube. Failure was defined as obstruction (reflux or no passage) at that visit. Irrigation was performed by the operating surgeon, who was aware of the postoperative protocol; outcome assessment was therefore not masked. Functional outcome was recorded at the same visit as the presence or absence of epiphora reported by the patient; no validated symptom score was used. Age (years), sex, and primary versus revision surgery were recorded; age and sex were used as covariates. Aetiology of the obstruction, duration of symptoms, previous episodes of dacryocystitis, previous nasal surgery, and nasal or systemic comorbidities were not systematically recorded in the clinical records and could therefore not be analysed.

### 2.6. Statistical Analysis

Continuous variables were assessed for normality with the Shapiro–Wilk test and are reported as median and interquartile range (IQR); they were compared with the Wilcoxon rank-sum test. Categorical variables are reported as counts and percentages and were compared with the chi-square test or Fisher’s exact test, as appropriate. Success rates are reported with Wilson 95% confidence intervals (CIs), and the absolute difference in success between groups with a Newcombe hybrid-score 95% CI. Because of the small number of failures, the primary comparison used Fisher’s exact test. To account for baseline imbalance, a multivariable logistic regression model was fitted with success (patent = 1, obstructed = 0) as the dependent variable and treatment group (mometasone as reference), age (per year), and sex (female as reference) as covariates; results are expressed as odds ratios (ORs) with 95% Wald CIs. The number of covariates was restricted to three to limit overfitting given the number of failure events.

As the sample size was fixed by the available records, a sensitivity analysis was performed to establish the smallest difference in success that the study could detect. With the observed group sizes (64 and 179), a two-sided α of 0.05, and a control-group success of 90%, the study had approximately 50% power to detect an absolute difference of 10 percentage points and approximately 80% power to detect a difference of 15 percentage points (pwr.2p2n.test, R package pwr). A two-sided *p* < 0.05 was considered statistically significant. Analyses were performed with R version 4.5.1 (R Foundation for Statistical Computing, Vienna, Austria). There were no missing data for the analysed variables.

## 3. Results

### 3.1. Participants

A total of 243 EXT-DCR procedures in 211 patients were analysed: 64 procedures (58 patients) in the mometasone group and 179 procedures (153 patients) in the control group. Baseline characteristics are shown in Table 1. Age was not normally distributed in either group (Shapiro–Wilk *p* < 0.001). The median age was 70.5 years (IQR 62.0–77.0; range 37–87) in the mometasone group and 73.0 years (IQR 65.5–79.0; range 33–91) in the control group (*p* = 0.23). The proportion of women was higher in the mometasone group (54/64, 84.4% vs. 119/179, 66.5%; χ^2^ = 6.54, *p* = 0.011). The proportion of revision procedures was similar in both groups (4/64, 6.3% vs. 11/179, 6.1%; Fisher’s exact test, *p* = 1.00).

### 3.2. Primary Outcome

**Figure 1:**
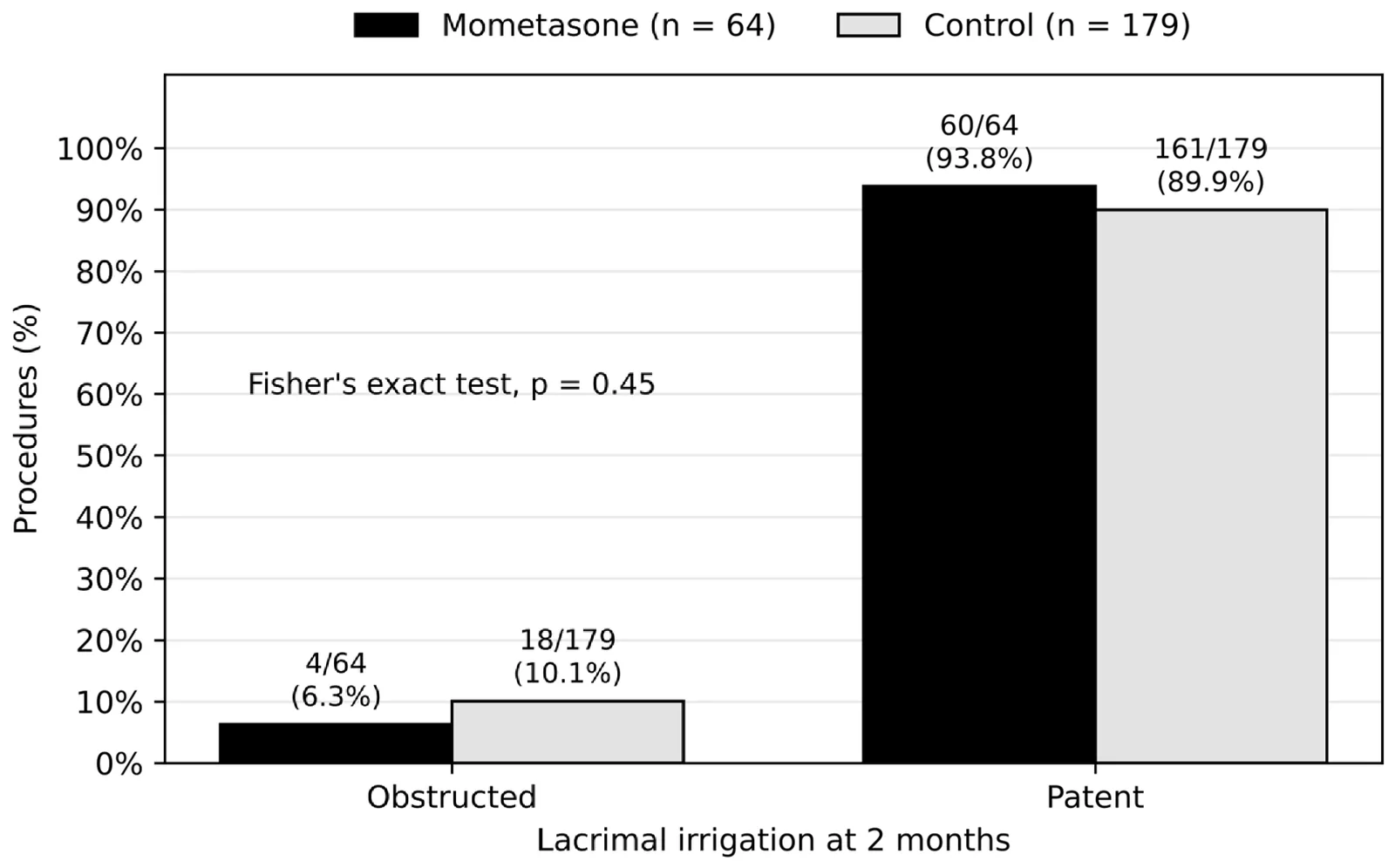
Anatomical outcome on lacrimal irrigation at two months by treatment group. Bars show the percentage of procedures with a patent or obstructed lacrimal pathway in the mometasone group (black) and the control group (grey); labels show *n*/N (%). Groups were compared with Fisher’s exact test.

Anatomical success at two months was achieved in 60 of 64 procedures (93.8%; 95% CI 85.0–97.5%) in the mometasone group and in 161 of 179 procedures (89.9%; 95% CI 84.7–93.5%) in the control group (Table 2). The absolute difference was 3.8 percentage points in favour of the mometasone group (95% CI −5.7 to 10.3), which was not statistically significant (Fisher’s exact test, *p* = 0.45) (Figure 1). The confidence interval indicates that the data are compatible with effects ranging from a 5.7-point disadvantage to a 10.3-point advantage for mometasone. All patients with a patent lacrimal pathway at two months reported resolution of epiphora.

### 3.3. Multivariable Analysis

In the multivariable logistic regression model, treatment group was not independently associated with anatomical success after adjustment for age and sex (control vs. mometasone: OR 0.57; 95% CI 0.18–1.77; *p* = 0.33). No statistically significant associations were detected for age (OR 0.99 per year; 95% CI 0.96–1.02; *p* = 0.55) or male sex (OR 1.55; 95% CI 0.55–4.40; *p* = 0.41) (Table 3). The wide confidence interval for the treatment effect reflects the small number of failures (22 of 243 procedures) and indicates considerable uncertainty about both the magnitude and the direction of any effect of mometasone.

## 4. Discussion

In this two-centre cohort of 243 EXT-DCR procedures performed by a single surgeon with a standardized technique, the postoperative protocol that added a two-month course of intranasal mometasone to standard postoperative care was not associated with a statistically significant improvement in early anatomical success (irrigation patency at two months). Success rates in both groups (93.8% and 89.9%) were consistent with the pooled success of EXT-DCR reported in the literature [2].

These findings must be interpreted in light of the precision of the estimate. The 95% CI for the difference ranged from a 5.7-point disadvantage to a 10.3-point advantage for mometasone. Our data are therefore compatible with no effect but cannot exclude a clinically meaningful benefit of up to approximately ten percentage points, and the absence of a statistically significant difference should not be read as evidence of equivalence. This is consistent with the sensitivity analysis, which showed adequate power only for differences of 15 points or more. Given that baseline success of EXT-DCR is around 90%, very large samples would be required to demonstrate small absolute benefits of any adjuvant therapy, which may explain why the literature on this topic remains inconclusive [6].

The available evidence on postoperative corticosteroids in DCR derives almost entirely from END-DCR. Kang et al. reported that triamcinolone-soaked nasal packing reduced the incidence of postoperative granulation (10.0% vs. 20.4%) and improved overall functional success, whereas the improvement in anatomical success did not reach statistical significance (96.0% vs. 92.7%) [11]. Similarly, Chen et al. found that methylprednisolone-soaked Spongostan placed over the rhinostomy significantly improved functional success (93.4% vs. 83.6%), whereas anatomical success did not differ significantly (94.6% vs. 90.9%) [13]. Kaptı and Korkmaz evaluated postoperative triamcinolone in END-DCR using a lacrimal symptom questionnaire [12], and Ma et al. described the gradual regression of ostium granulomas under continuous intranasal steroid treatment after END-DCR [14]. In the meta-analysis by Vinciguerra et al., nasal steroids did not significantly influence END-DCR outcomes (*p* = 0.58), and the effect of postoperative therapies after EXT-DCR could not be analysed for lack of data [6]. To our knowledge, the present study is among the first to compare postoperative protocols with and without intranasal corticosteroids after EXT-DCR, albeit indirectly through centre-specific protocols.

This study did not compare external and endoscopic DCR, and whether intranasal steroids have a smaller effect after EXT-DCR remains untested. A possible, but unproven, explanation relates to the way the ostium heals. In END-DCR, part of the nasal and lacrimal mucosa is removed and bare bone is often left exposed around the ostium, favouring secondary-intention healing, granulation tissue, and cicatricial narrowing [7,8]. In the external technique used here, anterior sac and nasal mucosal flaps are sutured together, creating a largely mucosa-lined anastomosis that heals by primary intention, with a silicone tube in place during the first two months. Under these conditions, it is conceivable that the anti-inflammatory and antifibrotic effect of a topical nasal steroid contributes less to ostium patency, although this hypothesis would require direct comparative study. Other adjuncts aimed at modulating wound healing, such as mitomycin C or 5-fluorouracil, have also shown uncertain benefit [17,18].

These findings have practical relevance. The regimen evaluated here—twice-daily application for two months—represents approximately 120 applications per patient, with associated cost, potential local adverse effects such as epistaxis or nasal dryness, and an additional burden on a predominantly elderly population. If confirmed by prospective studies, the omission of routine intranasal corticosteroids after uncomplicated EXT-DCR could simplify postoperative care without compromising outcomes. Until then, the benefit of this practice should be regarded as uncertain and evaluated rather than assumed.

Two aspects of the design deserve particular consideration when interpreting the treatment effect. First, treatment allocation was entirely determined by centre, so the comparison between groups is also a comparison between a private and a public centre. Patients treated in these two settings may differ in socioeconomic status, waiting time to surgery, duration and severity of the obstruction, and adherence to postoperative instructions, and the two cohorts were operated on over partly different periods (2021–2025 and 2019–2025). Because the mometasone cohort did not include procedures from the first two years of the study period, a learning-curve effect of the surgeon could have favoured this group. Adjustment for age and sex cannot account for these differences, and the direction of the resulting bias cannot be predicted with certainty. The proportion of revision procedures was similar in both groups, but other case-mix variables, such as nasal comorbidity, and the year-by-year distribution of procedures could not be compared. Our findings should therefore be interpreted as a comparison of two centre-specific postoperative protocols rather than as an estimate of the isolated pharmacological effect of mometasone. Second, anatomical success was assessed at two months, at the end of mometasone treatment and at the time of tube removal. Our results therefore reflect ostium patency during the period of steroid exposure, when any anti-inflammatory effect would be expected to be greatest, but they provide no information on late failures. Symptomatic failure after EXT-DCR may present many months after surgery—a median of 12 months was reported in a series of failed procedures [9]—and a late loss of patency after steroid withdrawal could not have been detected with our follow-up. The effect of intranasal mometasone on long-term patency therefore remains unknown, and our conclusions are restricted to early irrigation patency. Because symptoms were recorded only as the patient-reported presence or absence of epiphora, no conclusions can be drawn about the degree of symptom relief.

### Limitations

This study has several limitations. First, its retrospective, non-randomized design means that treatment allocation was determined by centre. Although the surgeon, technique, and remaining postoperative regimen were identical, centre-level differences in patient population (private vs. public healthcare setting), referral patterns, period of surgery, or perioperative care may have confounded the comparison, and this cannot be fully corrected by statistical adjustment. Second, the groups were unbalanced in size and in sex distribution; sex was included in the multivariable model, but residual confounding by unmeasured factors (e.g., aetiology, duration of obstruction, prior dacryocystitis, previous nasal surgery, or nasal and systemic comorbidity), which were not systematically recorded, cannot be excluded. Third, the outcome was assessed only at two months, coinciding with tube removal, and was limited to anatomical patency on irrigation; functional outcome was recorded only as the patient-reported presence or absence of epiphora, without a validated grading scale or symptom questionnaire, and endoscopic assessment of the ostium was not available; late failures occurring after this time point would not have been captured; long-term follow-up was not available for all patients and was therefore not analysed. Fourth, adherence to intranasal mometasone was not verified, and postoperative complications and adverse events were not analysed. In addition, irrigation was performed by the operating surgeon, who was aware of the protocol, so assessment bias cannot be excluded, although reflux on irrigation is a relatively objective endpoint. Fifth, the sample size allowed detection of large differences only. Sixth, 32 patients underwent staged bilateral surgery and contributed two procedures each; the possible correlation between fellow eyes was not accounted for in the analysis. The number of records screened and excluded was not recorded. Finally, all procedures were performed by a single experienced surgeon, which strengthens internal validity but may limit generalizability.

## 5. Conclusions

In this two-centre retrospective cohort, the postoperative protocol that included a two-month course of intranasal mometasone after EXT-DCR was not associated with a statistically significant improvement in early anatomical success (irrigation patency at two months) compared with the protocol without intranasal corticosteroids. Given the limited statistical power, the absence of a statistically significant difference does not establish therapeutic equivalence, and a clinically meaningful treatment effect cannot be definitively excluded. The effect of routine postoperative intranasal mometasone therefore remains uncertain, and an adequately powered randomized trial with long-term anatomical and functional outcomes is needed.

## Figures and Tables

**Table 1:** Baseline characteristics of the study groups.

Characteristic	Mometasone (*n* = 64)	Control (*n* = 179)	*p*-Value
Age, years, median (IQR)	70.5 (62.0–77.0)	73.0 (65.5–79.0)	0.23 ^a^
Age, years, range	37–87	33–91	—
Female sex, *n* (%)	54 (84.4)	119 (66.5)	0.011 ^b^
Male sex, *n* (%)	10 (15.6)	60 (33.5)	
Revision DCR, *n* (%)	4 (6.3)	11 (6.1)	1.00 ^c^
Patients, *n*	58	153	—
Patients with staged bilateral surgery, *n*	6	26	—
Centre (setting)	Clínica Baviera (private)	FOM (public)	—
Period of surgery	2021–2025	2019–2025	—

IQR, interquartile range. ^a^ Wilcoxon rank-sum test. ^b^ Chi-square test (χ^2^ = 6.54). ^c^ Fisher’s exact test. DCR, dacryocystorhinostomy.

**Table 2:** Anatomical success at two months by treatment group.

Outcome	Mometasone (*n* = 64)	Control (*n* = 179)	Difference (95% CI)
Patent, *n* (%)	60 (93.8)	161 (89.9)	3.8 pp (−5.7 to 10.3)
95% CI of success rate	85.0–97.5%	84.7–93.5%	
Obstructed, *n* (%)	4 (6.3)	18 (10.1)	
Fisher’s exact test			*p* = 0.45

pp, percentage points. Success rate CIs by the Wilson method; CI of the difference by the Newcombe hybrid-score method.

**Table 3:** Multivariable logistic regression for anatomical success (patent = 1).

Variable	OR	95% CI	*p*-Value
Group: control vs. mometasone	0.57	0.18–1.77	0.33
Age (per year)	0.99	0.96–1.02	0.55
Sex: male vs. female	1.55	0.55–4.40	0.41

OR, odds ratio; CI, confidence interval. Reference categories: mometasone group and female sex.

## Data Availability

The data presented in this study are available on request from the corresponding author due to patient privacy restrictions.
